# Long-Term Caloric Restriction Attenuates β-Amyloid Neuropathology and Is Accompanied by Autophagy in APPswe/PS1delta9 Mice

**DOI:** 10.3390/nu13030985

**Published:** 2021-03-18

**Authors:** Luisa Müller, Nicole Power Guerra, Jan Stenzel, Claire Rühlmann, Tobias Lindner, Bernd J. Krause, Brigitte Vollmar, Stefan Teipel, Angela Kuhla

**Affiliations:** 1 Rudolf-Zenker-Institute for Experimental Surgery, Medical University Rostock, 18057 Rostock, Germany; luisa.mueller2@uni-rostock.de (L.M.); nicole.guerra@uni-rostock.de (N.P.G.); Claire_ruehlmann@web.de (C.R.); brigitte.vollmar@med.uni-rostock.de (B.V.); 2 Department of Psychosomatic Medicine and Psychotherapy, University of Rostock, 18147 Rostock, Germany; stefan.teipel@med.uni-rostock.de; 3 Centre for Transdisciplinary Neurosciences Rostock (CTNR), University of Rostock, 18147 Rostock, Germany; 4 Core Facility Multimodal Small Animal Imaging, Rostock University Medical Center, 18057 Rostock, Germany; jan2.stenzel@gmail.com (J.S.); tobias.lindner@med.uni-rostock.de (T.L.); bernd.krause@med.uni-rostock.de (B.J.K.); 5 Department of Nuclear Medicine, Rostock University Medical Center, 18057 Rostock, Germany; 6 German Center for Neurodegenerative Diseases (DZNE)–Rostock/Greifswald, 18147 Rostock and 17489 Greifswald, Germany

**Keywords:** APPswe/PS1delta9, caloric restriction, [^18^F]FDG-PET/CT, amyloid β, iba1, autophagy

## Abstract

Caloric restriction (CR) slows the aging process, extends lifespan, and exerts neuroprotective effects. It is widely accepted that CR attenuates β-amyloid (Aβ) neuropathology in models of Alzheimer’s disease (AD) by so-far unknown mechanisms. One promising process induced by CR is autophagy, which is known to degrade aggregated proteins such as amyloids. In addition, autophagy positively regulates glucose uptake and may improve cerebral hypometabolism—a hallmark of AD—and, consequently, neural activity. To evaluate this hypothesis, APPswe/PS1delta9 (tg) mice and their littermates (wild-type, wt) underwent CR for either 16 or 68 weeks. Whereas short-term CR for 16 weeks revealed no noteworthy changes of AD phenotype in tg mice, long-term CR for 68 weeks showed beneficial effects. Thus, cerebral glucose metabolism and neuronal integrity were markedly increased upon 68 weeks CR in tg mice, indicated by an elevated hippocampal fluorodeoxyglucose [^18^F] ([^18^F]FDG) uptake and increased N-acetylaspartate-to-creatine ratio using positron emission tomography/computer tomography (PET/CT) imaging and magnet resonance spectroscopy (MRS). Improved neuronal activity and integrity resulted in a better cognitive performance within the Morris Water Maze. Moreover, CR for 68 weeks caused a significant increase of LC3BII and p62 protein expression, showing enhanced autophagy. Additionally, a significant decrease of Aβ plaques in tg mice in the hippocampus was observed, accompanied by reduced microgliosis as indicated by significantly decreased numbers of iba1-positive cells. In summary, long-term CR revealed an overall neuroprotective effect in tg mice. Further, this study shows, for the first time, that CR-induced autophagy in tg mice accompanies the observed attenuation of Aβ pathology.

## 1. Introduction

One hallmark of Alzheimer’s disease (AD) is the accumulation of amyloid-β (Aβ), leading to formation of Aβ-plaques [1]. Transgenic animal models of Aβ pathology provide mechanistic insight into aspects of AD pathology related to Aβ accumulation and represent an important tool for translational AD research. Accordingly, APPswe/PS1delta9 mice, a well-established AD mouse model, display a variety of clinically relevant AD-like symptoms, including increased parenchymal Aβ load, neuroinflammation, deficits in the cholinergic system, and cognitive impairment at an age of 4 months when developing first Aβ depositions [2]. Overall, neural activity in AD is linked with altered cerebral glucose metabolism. Accordingly, several clinical studies [3,4] have reported that hypometabolism is a well-described pathological hallmark of AD.

It is widely accepted that caloric intake may influence the relative risk for AD [5,6]. Most remarkably, while high caloric intake may promote AD neuropathology, experimental evidence strongly supports the hypothesis that caloric restriction (CR) prevents it. In this context, CR has attenuated Aβ deposition in several AD mouse models [7,8,9,10], whereby the duration of CR with 4 weeks [8] or 36 weeks [10] strongly differed. Moreover, Patel et al. and colleagues [8] showed reduced Aβ-associated astrocyte activation upon CR. Besides diminishing Aβ pathology, CR led to the reduction of neuronal loss in hippocampus [11] and to the improvement of cognitive deficits [12].

One process induced by CR is autophagy, a catabolic mechanism that degrades and recycles organelles and misfolded proteins such as Aβ. Therefore, autophagy is important in Aβ clearance from tissues [13]. Interestingly, AD-associated phenomena like neuroinflammation and glial activation can impair autophagy functionality, further amplifying neurodegeneration [14]. In this context, it has been reported that CR may induce glial autophagy, which is known to have a neuroprotective effect in AD [15]. In detail, autophagy is characterized by translocation of autophagy-gene-related (Atg) protein LC3BII together with sequestosom-1 (p62) to the autophagosome membrane, both commonly used as markers of autophagosome formation [16].

To date, no study has assessed whether CR-induced improvement in cognition is accompanied by autophagy. Moreover, the success of CR seems to be dependent on the duration of CR [17]. For example, our working group was able to show that only a lifelong CR for 74 weeks improved cognition performance in C57BL6 mice [18]. However, studies with AD mice have revealed an enhancement of cognition after 14 weeks CR [7,8]. To address this subject, we subjected APPswe/PS1delta9 mice to short-term (16 weeks) or long-term (68 weeks) CR and studied to what extent reduced amyloid pathology and improved cognition was accompanied with increased autophagy.

## 2. Materials and Methods

### 2.1. Animals

The study was performed in female APPswe/PS1delta9 (tg) mice co-expressing human amyloid-β precursor protein (APP) K594N and M595L mutation, as well as the human presenilin (PS) 1, L166P mutation under the control of the mouse prion protein promotor [19,20]. The APPswe/PS1delta9 mice were hemizygotes on B6xC3H and C57BL6 mouse backgrounds, and all mice were bred in-house. Female littermates of B6xC3H, as well as C57BL6 mice, were pooled and served as control group (wild-type; wt). Mice at the age of 4 weeks were fed either ad libitum (AL) or a caloric-restricted diet (CR, 60% of ad libitum chow) for 16 weeks (16 weeks or short-term, *n* = 5–10 for each group) or 68 weeks (68 weeks or long-term, *n* = 5–10 for each group). All mice were housed in standard cages in a temperature-controlled room (22 °C ± 2 °C) on a 12 h light/dark cycle (light on at 06:00 a.m.), with free access to water under specified pathogen-free conditions. At the beginning and end of the experiment, body weight was examined. Blood glucose was measured directly before sacrifice. The experimental protocol was approved by the local Animal Research Committee (Landesamt für Landwirtschaft, Lebensmittelsicherheit und Fischerei (LALLF) of the state Mecklenburg-Western Pomerania (LALLF M-V/TSD/7221.3-1.1-002/14)). All animals received care according to the German legislation on protection of animals and the Guide for the Care and Use of Laboratory Animals (European Directive 2010/63/EU).

### 2.2. Magnetic Resonance Imaging (MRI) and Spectroscopy (MRS)

**Figure 1:**
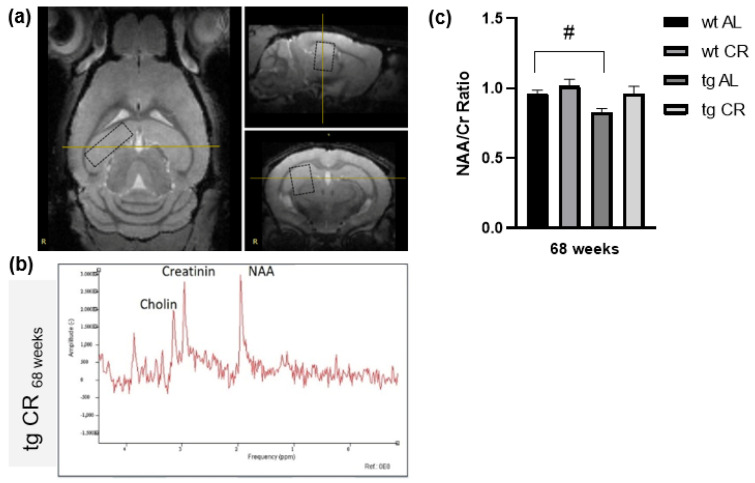
Representative magnetic resonance image including measured voxel (**a**). Example of magnetic resonance spectroscopy (MRS) with the prominent metabolites N-acetylaspartate (NAA resonates at 2.0 ppm) and creatine (Cr resonates at 3.0 ppm) from a transgenic APPswe/PS1delta9 (tg) mouse (**b**). Quantification of N-acetylaspartate/creatine (NAA/Cr) ratios in the brains of wild-type (wt) and tg mice fed either ad libitum (AL) or a caloric-restricted diet (CR, 60% of ad libitum) for 68 weeks (**c**). Values are given as mean ± SEM. Significance of differences between the groups was tested by one-way ANOVA on Ranks (Kruskal–Wallis) with Dunn’s post hoc test for multiple comparisons: # *p* < 0.05 vs. wt.

In vivo imaging (representative Figure 1a), as well as single-voxel spectroscopy, was performed according to the methodology described by Rühlmann et al. [21]. Spectra (exemplary in Figure 1b) were analyzed with jMRUI spectroscopy software (version 5.2) [22,23] and the jMRUI2XML package [24]. N-acetylaspartate/creatine ratios (NAA/Cr) were calculated. Therefore, the Hankel-Lanczos Singular Value Decomposition (HLSVD) method with 5 components was applied [25].

### 2.3. Positron Emission Tomography/Computer Tomography (PET/CT) Imaging and PET/CT-Data Analysis

PET/CT imaging and data analysis were performed according to previous works of our group [21,26,27] using PMOD software (version 3.7; PMOD Technologies LLC, Zürich, Switzerland). The processed PET images were subsequently co-registered with the mouse brain volume-of-interest (VOI) template (Mouse Mirrione atlas), and the PMOD software and tracer uptake values were extracted for each delineated VOI. Due to the fact that mice differed in body weight, the injected dose percentage per gram (ID%/g) was chosen as unit of measurement and acquired for each VOI.

### 2.4. Morris Water Maze Test

The Morris Water Maze (MWM) was performed as measure for spatial reference memory according to previously published work [18]. The amount of platform crosses, latency to first platform crossing, time spent on platform, and north (N)-quadrant crosses were monitored in real time by a video camera (15E objective, Computar, CBC Europe, Düsseldorf, Germany with Camera CCA1300-60gm, Basler AG, Ahrensburg, Germany), with subsequent digital analysis (Ethovision XT II.5, Noldus Information Technology, Wageningen, The Netherlands).

### 2.5. Sampling

At the end of the experiment, all mice were anesthetized with a mixture of ketamine (98 mg/kg bodyweight, medistar, Ascheberg, Germany) and xylazine (6.5 mg/kg bodyweight, Bayer, Leverkusen, Germany), exsanguinated by puncture of the vena cava inferior for immediate separation of plasma, and harvested of brain tissue.

### 2.6. Immunohistochemistry

**Figure A1:**
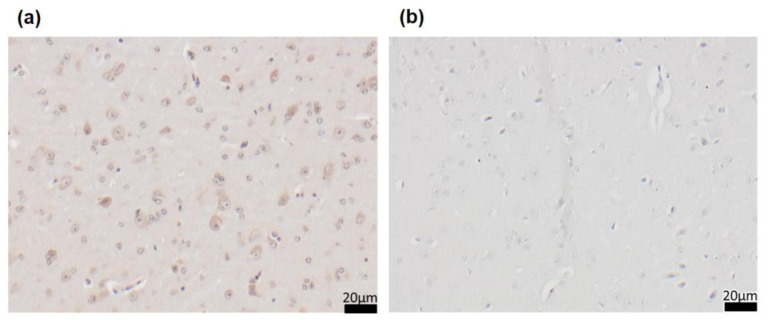
Negative controls of (**a**) 6E10 anti-Aβ staining and (**b**) iba1 staining.

Brain tissue was fixed in 4% phosphate-buffered formalin, embedded in paraffin and sliced in 4 µm-thin sections. The sections were put on X-tra Adhesive Precleaned Micro Slides (Leica, Wetzlar, Germany) and exposed to mouse monoclonal anti-Aβ antibody (clone 6E10; 1:1000, BioLegend, San Diego, CA, USA, as described by the authors of [28]) and goat polyclonal anti-iba1 antibody (1:1000, Abcam, Berlin, Germany). DAB chromogen Universal LSAB^®^ kits (System-HRP; DakoCytomation, Dako, Jena, Germany) were used for development according to the manufacturer’s instructions. The sections were counterstained with hemalaun (Merck, Darmstadt, Germany), and images were acquired on microscope type BX51 with a Color View Soft Imaging System and the corresponding software cellSens Standard 1.14 (all from Olympus, Hamburg, Germany). Appropriate negative staining images are provided in Appendix A (Figure A1). Within the hippocampus (*n* = 5–10 of each mouse strain and feeding), the number and the area of anti-Aβ positive plaques, as well as the number of iba1-positive cells, were assessed and measured semiautomatically with ImageJ 1.47 v. in a high-power field (HPF) and are given in n/HPF and µm^2^ for the area.

### 2.7. Western Blot Analysis of Brain Tissue

**Figure A2:**
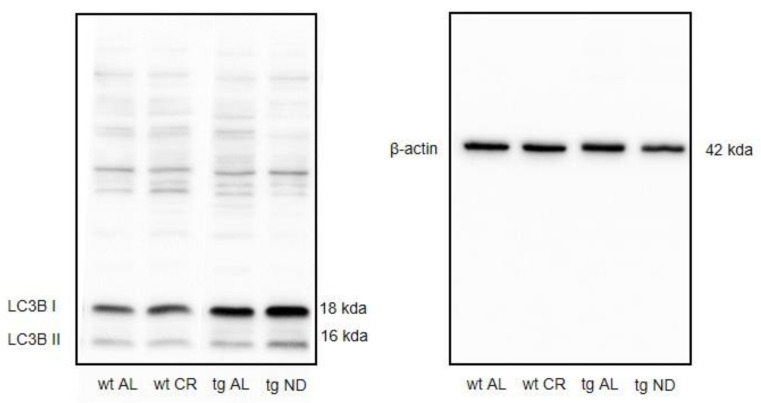
Raw Western blot:of LC3B and β-actin on 14% SDS gel.

**Figure A3:**
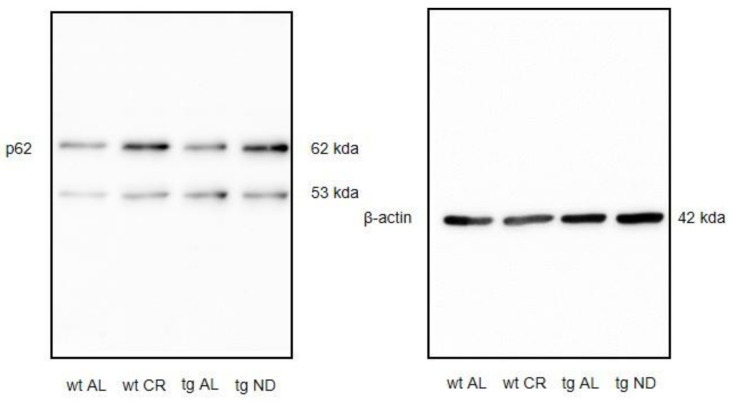
Raw Western blot of p62 and β-actin on 10% SDS gel.

Harvested brain tissue was further processed for protein isolation. For this purpose, brain tissue was homogenized in lysis buffer (10 mM Tris pH 7.5, 10 mM NaCl, 0.1 mM EDTA, 0.5% Triton-X 100, 0.02% NaN_3_ and 0.2 mM PMSF, protease inhibitor cocktail), incubated for 30 min on ice, and centrifuged for 10 min at 4 °C and 10,000× *g*. Protein contents were assayed by bicinchoninic acid method (Pierce Biotechnology, Rockford, IL, USA) with 2.5% BSA (Pierce Biotechnology, Rockford, IL, USA) as standard, as already described by the authors of [29]. On 14% (for LC3B) or 10% (for p62) SDS gels, 15 µg protein from brain tissue was separated and transferred to a polyvinyldifluoride membrane (Immobilon-P; Millipore, Burlington, MA, USA). After blockade with 2.5% BSA (Pierce Biotechnology, Rockford, IL, USA), membranes were incubated overnight at 4 °C with a rabbit polyclonal anti-p62 antibody (1:8000, Abcam, Berlin, Germany) and a rabbit polyclonal anti-LC3B antibody (1:1000, Sigma L7543, Sigma-Aldrich, Darmstadt, Germany). Exemplary raw images of western blot analysis of LC3B and p62 are shown in Appendix A (Figure A2 and Figure A3). The anti-LC3B antibody is able to detect both isoforms, LC3BI (~18 kDa) and LC3BII (~16 kDa), which can be distinguished by their corresponding molecular weight, as seen in the original blots (Appendix A, Figure A2). Afterward, a secondary HRP-linked anti-rabbit antibody (1:10,000, cell signaling 7074, Cell Signaling Technology, Frankfurt am Main, Germany) was applied. Visualization of protein expression was performed by means of luminol-enhanced chemiluminescence (ECL plus; Amersham Pharmacia Biotech, Amersham, UK). After digitalization with the ChemiDoc™ XRS System (Bio-Rad Laboratories, Hercules, CA, USA), signals were densitometrically assessed (Quantity One; Bio-Rad Laboratories, Hercules, CA, USA) and normalized to the density of β-actin signal in the respective specimen (acquired with mouse monoclonal anti-β-actin antibody; 1:20,000; Sigma, A5441 and secondary HRP-linked anti-mouse antibody; 1:60,000; Sigma, A9044, Sigma-Aldrich, Darmstadt, Germany).

### 2.8. Statistical Analysis

Data were checked for normal distribution with the Shapiro–Wilk test and variances of standard deviations were verified by Bartlett’s test. If standard deviations were not significantly different with *p* > 0.05, an ordinary one-way ANOVA was performed, followed by Sidak’s multiple comparisons test or unpaired student t-test followed by Bonferroni correction with *p* threshold of 0.0166 was performed. Otherwise, Brown–Forsythe and Welch ANOVA were performed followed by Tamhane’s T2 multiple comparisons test. If data were not normally distributed, the Kruskal–Wallis test with Dunn’s post hoc test for multiple comparisons or Mann–Whitney test, followed by Bonferroni correction with *p* threshold of 0.0166, was conducted. All data are expressed as mean ± standard error of mean (SEM). Statistical analysis was performed using the GraphPadPrism version 8.0.1 (GraphPad software, San Diego, CA, USA).

## 3. Results

### 3.1. Short-Term CR (16 Weeks) Showed No Effect in Glucose Uptake and Cognition Performance

CR vs. AL feeding for 16 weeks resulted in a significant decrease of bodyweight in wt and tg mice (*p* ≤ 0.0001, student t-test followed by Bonferroni correction, Table 1). Of utmost interest, blood sugar concentrations were almost unaffected by CR in both mouse strains (Table 1). In our study, short-term CR did not change [^18^F]FDG uptake (Table 2) nor cognitive performance (Table 3). Therefore, in the following evaluations, we only refer to the long-term CR.

### 3.2. Long-Term (68 Weeks) CR Significantly Increased [^18^F]FDG Uptake

**Figure 2:**
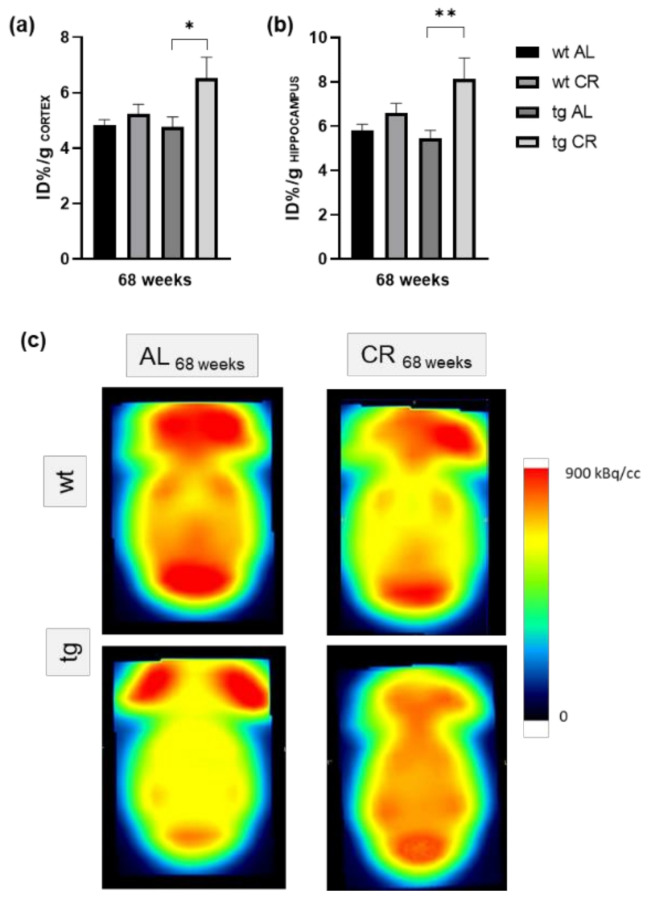
Quantification of [^18^F]FDG uptake in the cortex (**a**) and hippocampus (**b**) given as ID%/g of wild-type (wt) and transgenic APPswe/PS1delta9 (tg) mice. Mice were fed either ad libitum (AL) or a caloric-restricted (CR, 60% of ad libitum) diet for 68 weeks. Values are given as mean ± SEM. Significance of differences between the groups was tested by unpaired student t-test followed by Bonferroni correction with *p* threshold of 0.0166: * *p* < 0.05 or ** *p* < 0.005 vs. AL. Visual comparison of representative Images of [^18^F]FDG uptake in the brain of wt and tg mice (**c**).

^1^H-MRS demonstrated a significant reduction of the NAA/Cr ratio (*p* = 0.0272; Figure 1c) in tg vs. wt mice upon AL feeding. During CR feeding, the NAA/Cr ratio of tg mice tended to be elevated in contrast to AL-fed tg mice (*p* = 0.0523) and reached similar values as wt mice (Figure 1c). Whereas CR for 68 weeks caused significant reductions of approximately 30% of bodyweight in wt (*p* ≤ 0.0001, student t-test followed by Bonferroni correction) and tg mice (*p* = 0.0027, Mann–Whitney test followed by Bonferroni correction) vs. AL feeding, the blood sugar concentrations were almost unchanged (Table 1). Long-term CR vs. AL feeding in tg mice resulted in a significant increase of [^18^F]FDG uptake in the cortex (*p* = 0.0161, student t-test followed by Bonferroni correction, Figure 2a) and hippocampus (*p* = 0.0035, student t-test followed by Bonferroni correction, Figure 2b) with representative PET-CT images (Figure 2c).

### 3.3. Long-Term CR Increased Working Memory

**Figure 3:**
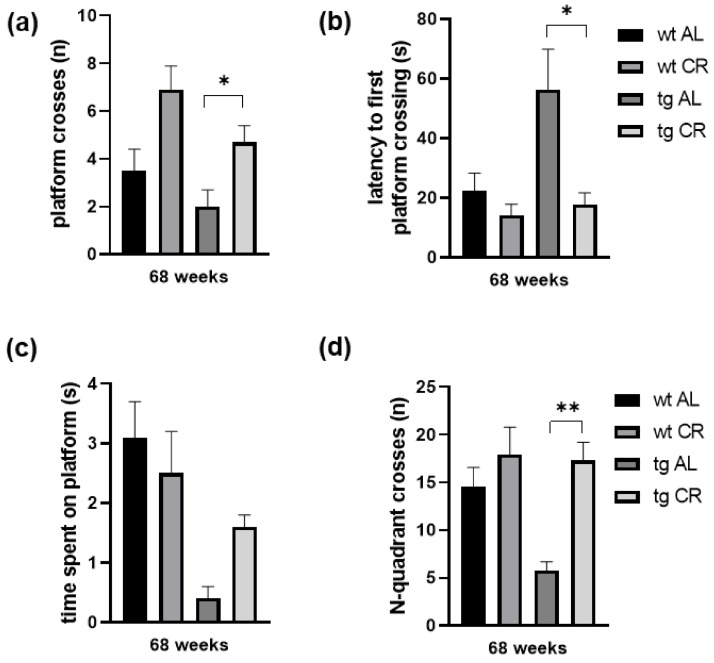
Number (*n*) of platform crosses during 60 s (**a**), latency (in s) to first platform crossing (**b**), time (in s) spent on platform (**c**), and n of north (N)-quadrant crosses (**d**) were measured for wild-type (wt) and transgenic APPswe/PS1delta9 (tg) mice. Mice were fed either ad libitum (AL) or a caloric-restricted diet (CR, 60% of ad libitum) for 68 weeks. Values are given as mean ± SEM. Significance of differences between the groups was tested by unpaired student *t*-test, followed by Bonferroni correction with *p* threshold of 0.0166: * *p* < 0.05 or ** *p* < 0.005 vs. AL.

Mice were tested in the spatial reference memory version of the MWM upon long-term CR. All mice were trained to find the platform (escape latencies were monitored, data not shown). In the trial, the platform was removed, and the number of platform crossings during 60 s was measured. The number of platform crosses measured in tg mice was almost half of those in wt mice upon AL feeding (Figure 3a). Long-term CR improved working memory performance as indicated by an almost two-fold increase of platform crosses in both mouse strains, whereas the increase was significant in tg mice (*p* = 0.0161, student t-test followed by Bonferroni correction Figure 3a). Moreover, latency to first platform crosses was found tendentially (up to three-fold) increased in tg mice when compared to wt mice. CR shortened latency in wt and significantly shortened latency in tg mice (*p* = 0.0069, student t-test followed by Bonferroni correction, Figure 3b). Correspondingly, time spent on platform was reduced up to eight-fold in tg vs. wt mice and was increased up to 4-fold upon long-term CR vs. AL in tg mice (Figure 3c). Additionally, the number of N-quadrant crosses was found tendentially decreased in tg vs. wt mice (*p* = 0.0200, student t-test followed by Bonferroni correction), and again significantly increased upon CR when compared to AL feeding (*p* = 0.0018, student t-test followed by Bonferroni correction; Figure 3d).

### 3.4. Long-Term CR Increased Autophagy

**Figure 4:**
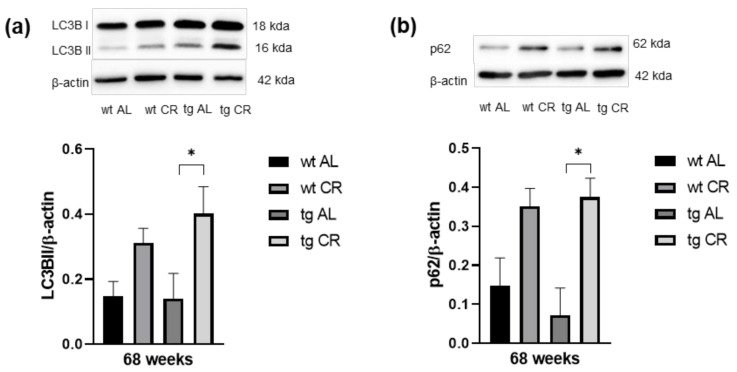
Representative Western blots, as well as densitometric analysis of (**a**) LC3BII and (**b**) p62 expression in brain of wild-type (wt) and transgenic APPswe/PS1delta9 (tg) mice. Mice were fed either ad libitum (AL) or a caloric-restricted diet (CR, 60% of ad libitum) for 68 weeks. Signals were corrected to that of β-actin. Values are given as mean ± SEM. Significance of differences between the groups was tested by one-way ANOVA on Ranks (Kruskal–Wallis) with Dunn’s post hoc test for multiple comparisons: * *p* < 0.05 vs. AL.

The analysis of LC3BII protein expression revealed no difference between AL-fed wt and tg mice, while CR caused a marked increase of LC3BII expression which was significant in tg mice (Figure 4a; *p* = 0.0392). The analysis of p62 protein expression revealed a slight decrease in AL-fed tg vs. wt mice (Figure 4b). Protein expression of p62 was markedly increased upon CR in both mouse strains but was increased more significantly in tg mice (*p* = 0.0176) (Figure 4b).

### 3.5. Long-Term CR Reduced Aβ-Plaque Load and Size as Well as Accompanying Neuroinflammation

**Figure 5:**
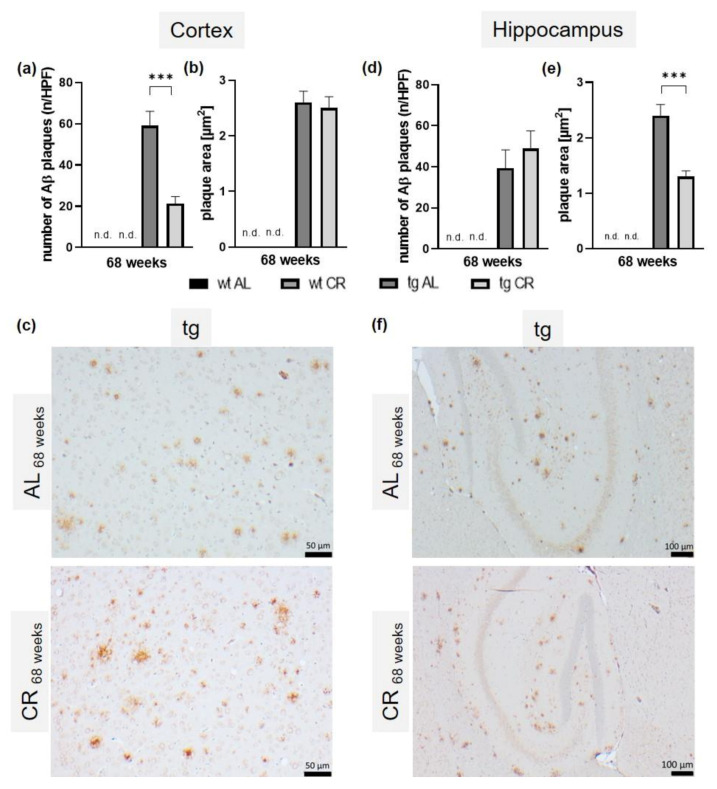
Quantitative analysis of the number of cortical (**a**) and hippocampal (**d**) amyloid-β (Aβ) plaques (*n*) per high-power field (HPF) and the cortical (**b**) and hippocampal (**e**) Aβ-plaque area (µm^2^) of wild-type (wt; not detectable n.d.) and transgenic APPswe/PS1delta9 (tg) mice and representative immunohistochemical images of cortical (scale bar representing 50 µm (**c**)) and hippocampal (scale bar representing 100 µm (**f**)) Aβ-stained (6E10) brain sections of tg mice. Mice were fed either ad libitum (AL) or a caloric-restricted diet (CR, 60% of ad libitum) for 68 weeks. Significance of differences between the groups was tested by unpaired student t-test, followed by Bonferroni correction with *p* threshold of 0.0166: *** *p* ≤ 0.001 vs. AL.

**Figure A4:**
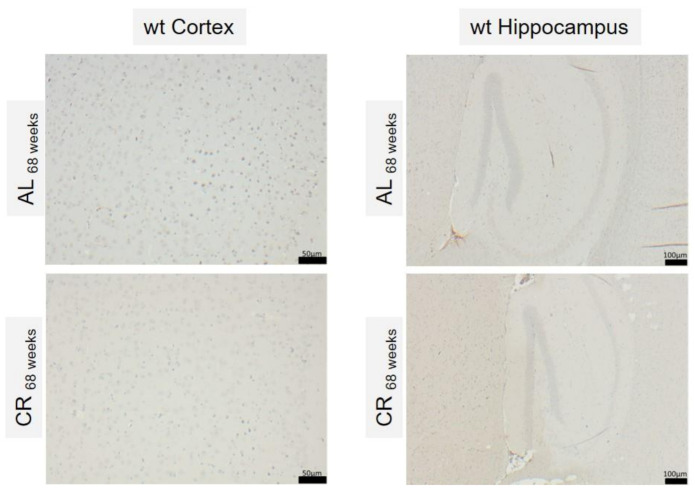
Representative immunohistochemical images of cortical (scale bar representing 50 µm) and hippocampal (scale bar representing 100 µm) Aβ-stained (6E10) brain sections of wt mice.

Analysis of Aβ-stained brain sections of tg mice (Figure 5a–f) revealed a mean plaque number of 39.5 ± 7.0 per HPF in the cortex (Figure 5a) and 59.1 ± 7.0 per HPF in the hippocampus (Figure 5d), and an average plaque size of 2.6 ± 0.2 µm2 in the cortex (Figure 5b) and 2.4 ± 0.2 µm^2^ in the hippocampus (Figure 5e). In the wt samples, no Aβ plaque could be detected (not detected, n.d.). Nevertheless, the respective images are provided in Appendix A (Figure A4). Long-term CR significantly reduced the Aβ plaque number (21.3 ± 3.4; *p* < 0.0001; student t-test followed by Bonferroni correction; Figure 5d) and plaque area (1.3 ± 0.1 µm^2^; *p* < 0.0001; student t-test followed by Bonferroni correction; Figure 5e) in the hippocampus.

**Figure 6:**
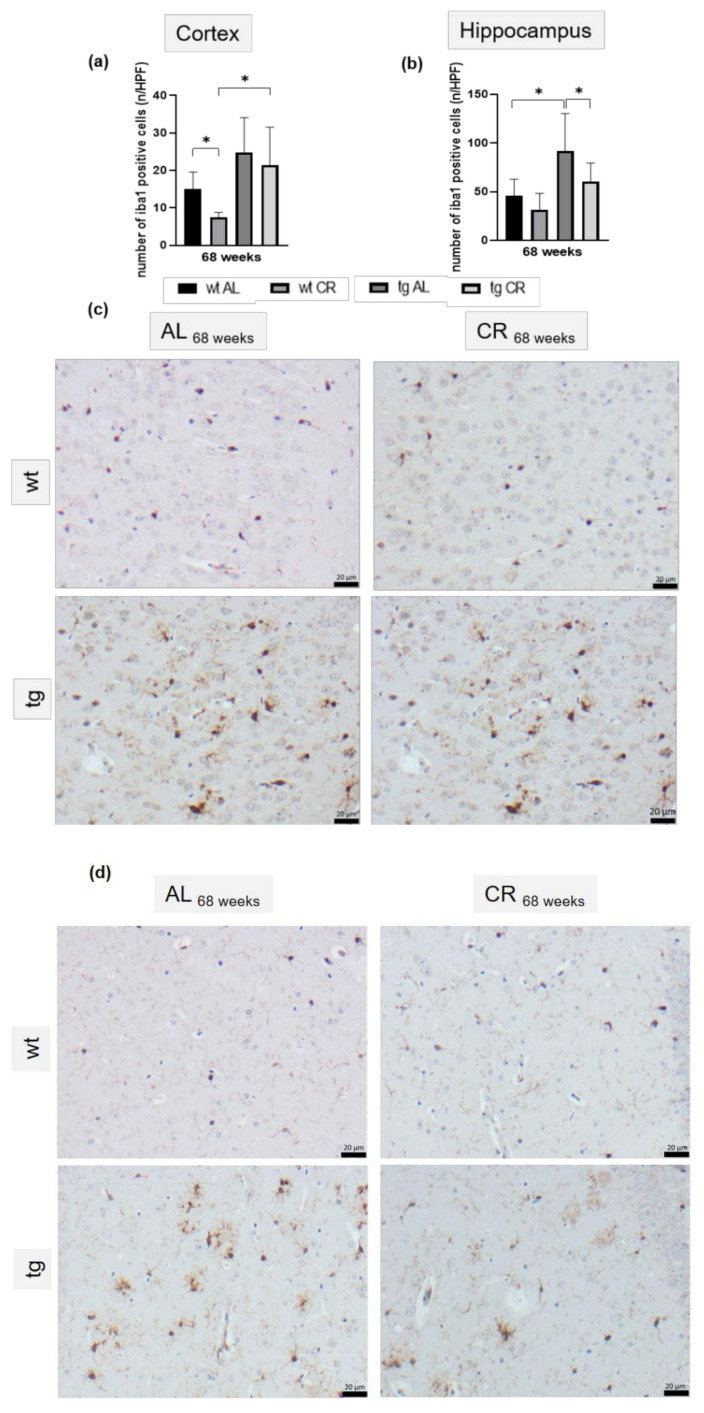
Quantitative analysis of the number of iba1-positive cortical (**a**) and hippocampal cells (**b**) per high-power field (*n*/HPF) of wild-type (wt) and transgenic APPswe/PS1delta9 (tg) mice and representative immunohistochemical images of cortical (**c**) and hippocampal (**d**) iba1-stained brain sections (scale bar representing 20 µm) of wt and tg mice. Mice were fed either ad libitum (AL) or a caloric-restricted diet (CR, 60% of ad libitum) for 68 weeks. Values are given as mean ± SEM; Significance of differences between the groups was tested by Brown–Forsythe and Welch ANOVA, followed by Tamhane’s T2 multiple comparisons test (**a**) or ordinary one-way ANOVA and Sidak’s multiple comparisons test (**b**): * *p* < 0.05.

The presence of plaque was accompanied by neuroinflammatory processes, displayed by a 40% increase of cortical (Figure 6a,b) and a significant increase of hippocampal (*p* = 0.0009; Figure 6c,d) iba1-positive cells in tg mice compared to wt mice. The reduction of plaque load and size in the hippocampus upon long-term CR in tg mice was accompanied by a significantly decreased number of iba1-positive hippocampal cells (*p* = 0.0329; Figure 6c).

## 4. Discussion

The main finding of the study was that CR ameliorates cognitive function by a measurable increase of glucose uptake, indicating a CR-induced increase in neuronal activity. This was accompanied by attenuated Aβ deposition and related microglia activation in the hippocampus. Overall, we speculate that this might be a result of CR-activated autophagy, but further investigation is needed to support this hypothesis.

CR-mediated attenuation of Aβ neuropathology in AD mouse studies has already been well described in literature. Mouton et al. [7] reported that CR reduced the total Aβ volume by about one-third in APP/PS1 mice. Moreover, Patel et al. [8] showed that, besides decreased plaque load and size, the immune-reactive area around Aβ plaques was markedly reduced upon CR in APP/PS1 mice as indicated by decreased numbers of astrocytes (GFAP-positive cells). Similarly, the current study demonstrates that the plaque load and size, as well as the number of activated microglia (iba1-positive cells), were significantly reduced in tg mice in the hippocampus. However, this could only be observed upon 68 weeks of CR. In contrast, 16-week ongoing CR showed no influence on Aβ pathology, although the study of Patel et al. [8] stated otherwise and was able to show an anti-amyloidogenic effect after 14 weeks of CR in double-transgenic APP/PS1 mice starting at 2 months of age. In contrast, longer CRs of 7 and 14 months were used by Halagappa et al. [12], showing that only CR of 14 months reduced Aβ levels and improved cognitive performance, as analyzed by MWM, in another transgenic mouse strain (3xTgAD) starting at 3 months of age. In this context, we are also able to show that only long-term CR attenuated the working memory of tg mice. Current literature has discussed whether better cognitive performance is mediated by CR-induced reduction of neuronal loss. Dong et al. [11] reported that CR significantly increased cell density in the CA3 region in the hippocampus. Moreover, CR has been described to prevent age-related disease or normal signs of age [30]. In this sense, the senescent (72-week-old) wt mice used in the current study benefited from low caloric intake, as indicated by better cognitive performance upon 68 weeks of CR. This anti-aging effect has been reported by several other groups [31,32,33]. In case of the AD mice, not only anti-aging processes are relevant for the neuroprotection, but anti-amyloidogenic mechanisms are also important [10]. Herein, it is shown that the mature form of ADAM10—an enzyme with α-secretase activity for the proteolytic processing of APP—was significantly upregulated in CR-fed Tg2576 mice [10], resulting in reduced Aβ plaque load. Since APPswe/PS1delta9 mice do not show any changes of ADAM10 expression in the hippocampus compared with their control littermates [34], we refrained from further investigating this pathway. Beside the anti-amyloidogenic process, CR is also able to induce autophagy, a well-documented catabolic mechanism which is known to degrade aggregated proteins [35], including Aβ. This processing pathway is characterized by translocation of Atg protein LC3BII, together with sequestosom-1 (p62) to the autophagosome membrane, which is commonly used as marker of autophagosome formation [16]. However, in AD animal models, autophagic activities have not yet been studied extensively as an underlying cause for the beneficial effects of CR [36]. Thus, it is still unclear if elevated autophagy has predominantly neuroprotective or neurodegenerative effects, as reported findings are partially contradictive. For example, CR-induced upregulation of SIRT-1 [37] may induce neuroprotective effects by upregulating autophagy through downstream signaling [36,38], whereas the neuroprotective effects of CR-upregulated BDNF [39] are due to autophagy suppression [40]. To complement the ambivalent data, the current study found that CR-fed tg mice exhibited a significant increase in autophagy. This was shown by elevated LC3BII and p62 levels, suggesting a neuroprotective mechanism of CR by restoration of cognitive function to wt levels through autophagy-induced Aβ degradation. Further experiments should be carried out in the future to clarify to what extend autophagy is the main driver of Aβ degradation.

Moreover, autophagy is also essential for accurate cellular and energy homeostasis. In this context, autophagy positively regulates glucose uptake via upregulation of GLUT-1 protein expression [41]. Therefore, it may be concluded that increased FDG uptake upon CR in tg mice is a consequence of increased autophagic activity. However, it can also be assumed that CR-induced autophagy and the associated Aβ degradation lead to improved neuronal activity per se, indicated by increased FDG uptake. Restrictively, it is known that reduced blood glucose concentration may enhance FDG uptake [42]. However, due to the barely changed blood glucose concentration upon CR, this does not seem to be the reason for the increased FDG uptake in CR-fed tg mice. In general, measurement of the effect of CR by [^18^F]FDG PET-CT opens the possibility to monitor CR-induced neuroprotection using a noninvasive method and, in particular, in a longitudinal manner. In addition to [^18^F]FDG-PET/CT, 1H-MRS represents another in vivo technique which allows for the characterization of metabolic changes in AD brains [43,44]. NAA, as a representative metabolite of neuronal integrity, is found to be reduced in AD, indicating neuronal malfunction either due to diminished neuronal density, neuronal cell loss, or partially reversible neuronal dysfunction [45], and correlates with disease progression [46]. Further, APP/PS1 mice also show significantly decreased NAA to Cr ratio [28,45,47], which was also observed in the present study. Upon long-term CR, NAA/Cr ratios were increased, reaching the same values as those found in wt mice, indicating improved neuronal integrity. However, the transfer of findings from transgenic animal models to humans is limited [48]. Nevertheless, the use of quantitative neuroimaging methods possibly aids the improvement of translational potential of preclinical AD research regarding brain metabolism or morphology [49].

## 5. Conclusions

In summary, the present study showed, for the first time, that the known CR-induced Aβ degradation [7,8] is accompanied by increased autophagy and improved neuronal activity as well as integrity, resulting in a better cognitive performance. Further studies need to clarify to what extent the observed increased autophagy in CR tg mice (upon 68 weeks CR) is responsible for the attenuation of the Aβ pathology.

## Figures and Tables

**Table 1:** Blood glucose concentrations and body weight of short- (16 weeks) and long-term (68 weeks) ad libitum (AL) or caloric-restricted (CR, 60% of ad libitum) diet-fed wild-type (wt) and transgenic (tg) mice (all mice revealed starting weight of approximately 19 g). Values are given as mean ± SEM. Significance of differences between the groups was tested by unpaired student t-test or Mann–Whitney test, both followed by Bonferroni correction with *p* threshold of 0.0166: ** *p* < 0.005; *** *p* ≤ 0.0001 vs. AL.

Genotype	wt		tg	
Feeding for 16 Weeks	AL	CR	AL	CR
Blood glucose (mmol/L)	7.94 ± 0.54	** 5.62 ± 0.27	7.15 ± 0.25	5.70 ± 0.27
Body weight (g)	28.90 ± 3.42	*** 20.26 ± 0.47	31.35 ± 0.95	*** 20.62 ± 0.44
**Feeding for 68 Weeks**	**AL**	**CR**	**AL**	**CR**
Blood glucose (mmol/L)	6.80 ± 0.27	6.78 ± 0.34	7.55 ± 0.05	6.15 ± 0.34
Body weight (g)	33.72 ± 2.29	*** 23.15 ± 0.55	28.88 ± 2.65	** 21.95 ± 0.71

**Table 2:** [^18^F]FDG uptake (ID%/g) measurements of short-term (16 weeks) ad libitum (AL) or caloric restricted (CR, 60% of ad libitum) diet-fed wild-type (wt) and transgenic (tg) mice. Values are given as mean ± SEM. Significance of differences between the groups was tested by Brown–Forsythe and Welch ANOVA, followed by Tamhane’s T2 multiple comparisons test (cortex) or ordinary one-way ANOVA and Sidak’s multiple comparisons test (hippocampus).

Genotype	wt		tg	
Feeding for 16 Weeks	AL	CR	AL	CR
[^18^F]FDG uptake (ID%/g)				
cortex	4.65 ± 0.25	4.90 ± 0.20	5.35 ± 0.25	5.18 ± 0.33
hippocampus	5.42 ± 0.28	5.46 ± 0.22	6.19 ± 0.31	5.94 ± 0.38

**Table 3:** Morris Water Maze parameters of short-term (16 weeks) ad libitum (AL) or caloric restricted (CR, 60% of ad libitum) diet-fed wild-type (wt) and transgenic (tg) mice. Values are given as mean ± SEM. Significance of differences between the groups was tested by unpaired student t-test, followed by Bonferroni correction with *p* threshold of 0.0166.

Genotype	wt		tg	
Feeding for 16 Weeks	AL	CR	AL	CR
Platform crosses (n)	6.0 ± 1.0	7.4 ± 1.5	5.0 ± 1.0	3.8 ± 0.9
Latency to first platform crossing (s)	10.1 ± 5.5	12.6 ± 4.2	37.7 ± 12.4	27.0 ± 7.1
Time spent on platform (s)	1.6 ± 0.5	2.9 ± 0.6	1.6 ± 0.6	1.5 ± 0.2
N-quadrant crosses (n)	18.3 ± 2.7	20.0 ± 2.3	19.5 ± 1.5	14.4 ± 1.2

## Data Availability

The data presented in this study are available on request from the corresponding author.
